# Chess Players Increase the Theta Power Spectrum When the Difficulty of the Opponent Increases: An EEG Study

**DOI:** 10.3390/ijerph17010046

**Published:** 2019-12-19

**Authors:** Juan Pedro Fuentes-García, Santos Villafaina, Daniel Collado-Mateo, Ricardo Cano-Plasencia, Narcis Gusi

**Affiliations:** 1 Faculty of Sport Science, University of Extremadura, Avda: Universidad S/N, 10003 Cáceres, Spain; jpfuent@unex.es (J.P.F.-G.); ngusi@unex.es (N.G.); 2 Centre for Sport Studies, Rey Juan Carlos University, 28943 Fuenlabrada, Spain; danicolladom@gmail.com; 3 Clinical Neurophysiology, San Pedro de Alcántara Hospital, 10003 Cáceres, Spain; ricanopla@hotmail.com

**Keywords:** brain, EEG power spectrum, chess, neural efficiency hypothesis

## Abstract

The present study aimed to analyze differences in the electroencephalogram (EEG) power spectrum (theta, alpha, and beta) between participants who won (winning group) and those who lost (losing group) in three different chess games: against their same Elo (100% chess games), 25% over their Elo (125% chess games), and 25% under their Elo (75% chess games). EEG was assessed at baseline and during the chess games. Method: 14 male chess players (age: 35.36 ± 13.77 and Elo: 1921 ± 170) played three games of 3 min, plus two additional seconds per move, while EEG was assessed. There were three difficulty levels (75%, 100%, and 125%), with two games (one with white pieces and another with black pieces) per level. The winning group showed higher theta power in the frontal, central, and posterior brain regions when difficulty increased (*p*-value < 0.05). Besides this, alpha power showed higher values (*p*-value < 0.05) in 125% games than in 75% chess games in C3, T3, T4, T5, and T6. The losing group showed a significant decrease (*p*-value < 0.05) in the beta and alpha power spectrum in frontal, central, parietotemporal, and occipital areas, when the opponent’s difficulty increased. Moreover, between groups, analyses showed higher theta power in the losing group than in the winning group, in C3, T5, T6, P4, and Pz (*p*-value < 0.05). Therefore, the winning group was able to adapt to each difficulty level, increasing theta power in the frontal, central, and posterior brain areas, as the efficiency hypothesis postulated. These changes were not observed in the losing group. Moreover, increases in alpha power during the most difficult games, in comparison with the easier, could have been caused by creative ideation and divergent thinking, as participants looked for alternative solutions against a higher-skilled opponent.

## 1. Introduction

The game of chess offers an excellent opportunity for the study of cognitive processes such as memory, perception, decision-making, and problem-solving [1]. Moreover, a recent meta-analysis showed that chess players outperformed non-chess players in intelligence-related skills [2]. Thus, researchers have focused on investigating mental load or activation patterns during different chess modalities, such as solving chess problems [3] where chess players showed activation of frontal, temporal, parietal, and occipital areas [4,5,6]. In addition, previous researchers have studied the impact of cognitive load in the autonomic modulation or electroencephalographic (EEG) signal [7,8,9,10,11].

Furthermore, expertise paradigms have been explored in chess. Different studies have shown differences between experts and novices in evoked EEG coherence, showing that experts have higher task-related functional integration of the cortical areas, [3] superior pattern recognition, and memory retrieval of chunks [12]. However, to our knowledge, differences in cortical activation between winners and losers during chess games have not been studied.

EEG has been commonly used to study cognitive processes of the brain. Different results have been obtained in the study of EEG power in the different power spectrum bands. In this regard, the theta power spectrum (4–7 Hz) increased when task difficulty increased, or when higher levels of mental effort were required [13,14,15,16,17]. For that reason, theta has been considered a potential indicator of cognitive effort or could be an indicator of success in cognitive tasks [18]. The alpha band (8–12 Hz) decreased during arithmetic tasks [15] and increased during creative thinking in prefrontal and parietal cortical areas [19]. Also, studies have found that right parietal alpha and beta (13–30 Hz) activity decreased in inverse relation to task difficulty [20]. Furthermore, the beta power spectrum serves as the indicator of behavioral arousal and attentional process [21].

Previous studies in chess have evaluated the psychophysiological response in different situations in order to study the stress or decision-making process among others [7,8,9,10,11,22]. However, the psychophysiological response of chess players who win or lose has never been explored. Therefore, the present study aims to investigate the differences in the EEG power spectrum in three different frequency bands (theta, alpha, and beta) between two groups (winning and losing) during chess games at three difficulty levels. Our hypothesis can be divided into three. First, there will be differences between those who win (winning group) and those who lose (losing group), as observed by a previous study [18]. Second, we hypothesize that theta power will increase when task difficulty increases [11,16]. Lastly, alpha and beta activity will decrease when task difficulty increases [15].

## 2. Materials and Methods

### 2.1. Participants

A total of 14 right-handed male chess players (age = 35.36 ± 13.77) were analyzed. Participants gave written, informed consent to participate in the study. All procedures were approved by the University research ethics committee (approval number: 85/2015), according to the Declaration of Helsinki. Exclusion criteria included: (a) inability to perform the tasks with the computer; (b) diseases or medication that affect the nervous system; and (c) not being classified by the International Chess Federation with Elo. The playing level for chess is determined by the Elo rating system, developed by Arpad Elo [23], and introduced by the International Chess Federation (FIDE). It is a method for calculating the relative skill levels of players in competitor-versus-competitor games [24]. Participants in the present study had a mean Elo of 1921.07 ± 170.67.

Volunteers were classified according to their results in the chess games. A victory was recompensed with 0.5 points and a stalemate with 0.25 points. Since there were six games (each level of difficulty was played with both black and white pieces), the maximal score was 3. Those players who obtained 1.5 or more made up the winning group, and the rest of the participants were included in the losing group.

### 2.2. Procedure

Before starting the study, participants received instructions about the procedures and protocol requirements during the chess games. All participants underwent a familiarization period with the computer and the equipment that was required for testing. The experimental room was calm, and light and temperature were continuously regulated.

Participants conducted three chess games of 3 min, plus 2 s of additional time per move, starting from move one. Each chess game had both black and white pieces. There were three difficulty levels: against their same Elo (100% chess games), 25% over their Elo (125% chess games), and 25% under their Elo (75% chess games). The order of tasks was randomized, and controlled, in order to avoid the effects of one task on other.

Chess games were developed with Fritz 15 software, using Stockfish 6, 64-BIT, as a chess engine that has been previously analyzed [25]. It is one of the most robust chess engines in the world, and it is open source, with a General Public License (GPL). Chess engines are a useful tool for chess training, reproducing the tactical responses of humans [26]. An ASUS laptop was employed (Intel Core i7-6500U, 1 TB, 8 GB memory, DDR3L-SDRAM). Fritz software responded automatically to moves, simulating a real chess environment. The research technician selected the Fritz level according to the Elo level of each player (the skill level of each chess player), setting the appropriate difficulty (75%, 100%, or 125% of their Elo). This allowed for the control and regulation of the skill level of the opponents and, consequently, control of the cognitive loads in the chess players.

### 2.3. Instruments and Outcomes

EEG data were assessed using the Enobio device, a wireless electrode system (Neuroelectrics, Cambridge, MA, USA) (Ruffini et al. 2006, 2007). The reliability of this instrument was demonstrated, even using dry electrodes [27].

EEG was recorded from 17 scalp locations, including: frontal (Fz, F3, F4, F7, and F8), central (Cz, C3, and C4), temporal (T3, T4, T5, and T6), parietal (Pz, P3, and P4), and occipital (O1 and O2), according to the International 10–20 system.

Electrodes placed in the mastoids served as references. A sampling rate of 500 Hz was used. The EEGLAB toolbox (MATLAB) was utilized for preprocessing and data analysis. A 50 Hz notch filter and bandpass filter (0.1–30 Hz) were employed and eye movement artifacts were corrected using an independent component analysis (ICA) [28]. Spectral analyses were performed for each chess game’s level of difficulty using MATLAB. The data were banded into theta (4–8 Hz), alpha (8–12 Hz), and beta (13–29 Hz) frequency bands.

### 2.4. Statistical Analysis

EEG data were normalized, dividing by the power spectrum at baseline. The Statistical Package for Social Sciences (SPSS) (version 20.0; SPSS, Inc., Chicago, IL, USA) was used to analyze the data.

The Shapiro–Wilk test was used to determine whether the distribution of variables was normal. Normal distribution was not assumed for all variables since the *p*-value was <0.05. Wilcoxon signed-rank tests were conducted to examine differences within groups in the chess games. Moreover, EEG data were analyzed with a Mann–Whitney U test to explore differences between groups in chess games. The alpha level of significance was set at 0.05.

## 3. Results

### 3.1. Theta Power Spectrum

Group differences, when both groups played against different levels of difficulty, were analyzed using the Wilcoxon signed-rank test. In the winning group, theta power was higher when the opponent’s difficulty increased. Comparing brain activity in the theta band, at 75% and 100% of the Elo, the channels F3 (*p* = 0.04), Fz (*p* = 0.03), F7 (*p* = 0.04), C3 (*p* = 0.03), Cz (*p* = 0.04), C4 (*p* = 0.03), Pz (*p* = 0.04), P4 (*p* = 0.03), T5 (*p* = 0.03), T6 (*p* = 0.04), T8 (*p* = 0.03), and O1 (*p* = 0.03) were higher when the opponent was difficult (see Table 1).

This same relation was observed when comparing 75% versus 125% chess games in high-performance groups in theta power, being higher values when the difficulty of the opponent increased in F3 (*p* = 0.04), F7 (*p* = 0.04), Cz (*p* = 0.04), C4 (*p* = 0.04), Pz (*p* = 0.04), P4 (*p* = 0.04), T5 (*p* = 0.03), T6 (*p* = 0.04), T8 (*p* = 0.03), and O1 (*p* = 0.04) (see Table 1).

Besides this, theta band in F3 and F4 values increased in the 125% chess game, compared to the 100% chess game (see Table 1).

### 3.2. Alpha and Beta Power Spectrum

In the losing group, the Wilcoxon signed-rank test showed that beta power decreased when the opponent’s Elo was higher. Thus, beta power, playing at 75% in comparison with 100%, was higher in the Fz, F7, F8, C3, Cz, C4, P3, Pz, P4, T5, T6, T8, O1, and O2 scalp locations. Moreover, these same effects were found in alpha power in the C3, Cz, C4, P3, Pz, P4, T5, T6, T8, O1, and O2 scalp locations (see Table 2).

However, when the winning group was analyzed, significant effects of difficulty were not observed in the beta band. In addition, alpha power showed higher values in the 125% chess game than in the 75% chess game in C3, T3, T4, T5, and T6 (see Table 2).

### 3.3. Task Comparison

**Figure 1:**
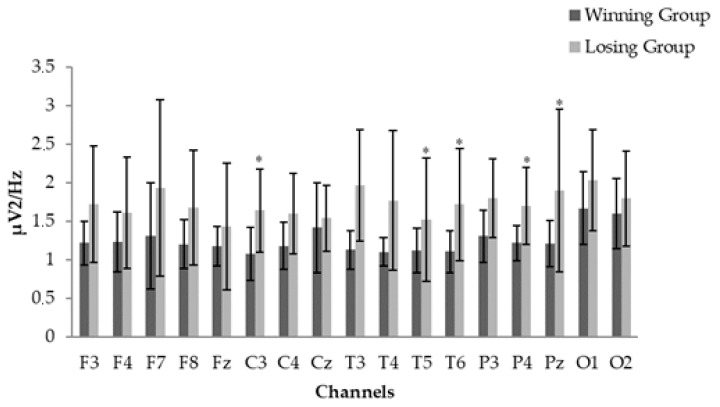
Channel comparison between groups in −25% chess games in the theta power spectrum. The Mann–Whitney test was conducted. Bars represent the standard deviation of the mean. *: Significant higher values (*p* < 0.05) between winning versus losing groups.

Differences between winning and losing groups playing at 75% of their Elo were calculated using the Mann–Whitney U test. Theta power in C3 (*p* = 0.02), T5 (*p* = 0.01), T6 (*p* = 0.02), P4 (*p* = 0.03), and Pz (*p* = 0.03), were higher in the losing group than in the winning group (see Figure 1). Other significances between group differences were not found in the rest of the power bands or chess games.

## 4. Discussion

The present study investigated, for the first time, the differences in EEG power spectrum in frequency bands (theta, alpha, and beta) between those players who won and those who lost three different chess games at 75%, 100%, and 125% of their Elo. Analyzing the results, increments in theta power were higher when the opponent’s difficulty increased in the winning group, but not in the losing group. Also, alpha and beta power in the losing group decreased when the difficulty of the opponent increased. This relation was not observed in the winning group, which showed higher alpha power in posterior regions in the game at 125% in 75% of chess games. Furthermore, different cortical activation between groups was found. Theta power was higher in the losing group than in the winning group in 75% of chess games.

Our results showed differences in the cortical activation pattern between participants who won and those who lost in the same tasks. Increases in theta power, when difficulty increased, have been previously reported [14,15,29]. In the same way, when comparing two performance groups, Zakrzewska and Brzezicka [30] found that the more difficult the working memory tasks, the greater the theta power. A previous study of adolescent chess players showed increments in the theta power spectrum when the difficulty of the task was raised [11]. In the present study, the winning group was able to adapt to each chess game situation, increasing theta power when the opponent’s difficulty increased. Nevertheless, these adaptations were not observed in the losing group. This could be explained by the neural efficiency hypothesis, which describes how brighter individuals show lower brain activation than less bright individuals when working on the same cognitive tasks, having more efficient neural networks [31].

Accordingly, group differences were found in chess games when playing against an opponent with a lower Elo. Better performance was obtained with the winning group, despite showing lower theta power than the losing group. These differences could be explained because chess games might have imposed less cognitive load on the winning group than on the losing group. Taken together, these results could also be supported by the neural efficiency hypothesis [31].

In addition, decreases in alpha and beta power were found in the losing group when playing against a more difficult opponent (75% vs. 100%) in chess games, and no differences were observed between 100% and 125%. Decreases in these power spectrum bands are related to increases in cognitive effort [15]. Thus, cognitive effort was significantly increased in the losing group when the difficulty of the task changed from 75% to 100% of their Elo. However, given that there were no differences between the 100% and the 125% tasks, it could be hypothesized that these two difficulties were an overcome stimulus, and participants from that group were not able to adapt to it. Similarly, a previous study showed that chess players with a low performance in problem-solving tasks did not adjust their autonomic modulation (heart rate variability) to an overcome stimulus while the high performance group did.

Furthermore, increased alpha power was found in posterior regions in the winning group between 75% and 125% of chess games. Increased alpha power has been reported during creative ideation and divergent thinking [19,32]. These findings suggest that the winning group could try to solve a difficult cognitive stimulus through divergent thinking and creativity, by trying to look for alternative solutions. However, further studies on this topic are necessary.

Our results could have significant applications in the field of chess training and health. In this regard, to our knowledge, this is the first study that shows a different psychophysiological response between chess players who win and those who lose when the difficulty of the opponent increases. Thus, this frequency band may be useful to control the cognitive load in chess training. In addition, theta power monitoring (4–8 Hz) could be incorporated into cognitive rehabilitation programs to control the stimulus caused by cognitive tasks, creating programs that are more efficient. This could be the case of neurofeedback intervention, which aims to be a feasible tool for therapeutic interventions and cognitive enhancement [33]. Among others, neurofeedback interventions have been proven to be a therapy for fibromyalgia patients, improving quality of life, psychological symptoms, and pain [34]. Nevertheless, future studies should investigate nonlinear parameters, such as entropy, which could be highly useful when studying different mental states [35].

One potential limitation might be that movements in chess games were conducted using a computer. Although chess engines are a common tool for chess training [26], chess players usually prefer playing on chess boards. Chess players who were not familiarized with playing chess with a computer could find it more difficult than playing on a chessboard. Therefore, in this study, all participants had previously played chess with the computer in order to avoid problems with familiarization. Lastly, the relatively small sample size (*n* = 14) might have caused that only great difference, having reached the statistical significance level. Therefore, further studies on this topic are encouraged to use higher sample sizes and also include nonlinear analyses.

## 5. Conclusions

Participants who won their games were able to adapt to each chess game situation, increasing theta power when the opponent’s difficulty increased. Also, participants who lost showed higher theta power than those who won, which could be supported by the neural efficiency hypothesis. In addition, decreased alpha and beta power were found in the losing group between chess games at 75% and 100% of their Elo, but not between games at 100% and 125%, which may be related to an incapacity to adapt to the higher levels of difficulty. Increased alpha power was found in posterior regions in the winning group between chess games at 75% and 125% of their Elo, which could have been caused by creative ideation and divergent thinking while looking for alternative solutions against a difficult opponent. However, results must be taken with caution, given the small sample size, and further studies are needed to confirm these results.

## Figures and Tables

**Table 1:** Theta power evolution in 3 + 2 chess games in winning and losing groups.

	Winning Group			Losing Group		
Channels	75% vs. 100%	75% vs. 125%	100% vs. 125%	75% vs. 100%	75% vs. 125%	100% vs. 125%
Electroencephalographic (EEG) Channels						
F3	0.04 ^b^	0.04 ^b^	0.04 ^b^	0.33	0.67	0.33
Fz	0.03 ^b^	0.35	0.46	0.67	0.48	0.78
F4	0.35	0.35	0.04 ^b^	0.48	0.58	0.78
F7	0.04 ^b^	0.04 ^b^	0.08	0.07	0.40	0.21
C3	0.03 ^b^	0.12	0.75	0.58	0.40	1.00
Cz	0.04 ^b^	0.04 ^b^	0.89	0.21	0.40	0.78
C4	0.03 ^b^	0.04 ^b^	0.92	0.16	0.58	0.58
P3	0.35	0.44	0.92	0.02 ^a^	0.07	0.80
Pz	0.04 ^b^	0.12	0.60	0.21	0.12	1.00
P4	0.03 ^b^	0.04 ^b^	0.75	0.18	0.13	0.61
T5	0.03 ^b^	0.03 ^b^	0.60	0.40	0.12	1.00
T6	0.04 ^b^	0.04 ^b^	0.46	0.12	0.48	0.58
T7	0.17	0.04 ^b^	0.46	0.67	0.67	0.67
T8	0.03 ^b^	0.03 ^b^	0.35	0.58	0.58	0.78
O1	0.03 ^b^	0.04 ^b^	0.12	0.21	0.26	0.78

^a^ Significantly higher values obtained against the easier opponent; ^b^ Significantly higher values obtained against the most difficult opponent.

**Table 2:** Between chess game differences in the alpha and beta power band in the losing and winning groups.

Channels	Low Performance		High Performance	
Chess games	−75% vs. 100%		125% vs. −75%	
	**Beta**	**Alpha**	**Beta**	**Alpha**
Fz	0.03 ^a^	Ns	NS	NS
F7	0.03 ^a^	Ns	NS	NS
F8	0.02 ^a^	Ns	NS	NS
C3	0.02 ^a^	0.03 ^a^	NS	0.03 ^b^
Cz	0.02 ^a^	0.03 ^a^	NS	NS
C4	0.02 ^a^	0.04 ^a^	NS	NS
P3	0.02 ^a^	0.01 ^a^	NS	NS
Pz	0.02 ^a^	0.02 ^a^	NS	NS
P4	0.02 ^a^	0.03 ^a^	NS	NS
T3	NS	NS	NS	0.03 ^b^
T4	NS	NS	NS	0.03 ^b^
T5	0.03 ^a^	0.02 ^a^	NS	0.03 ^b^
T6	0.02 ^a^	0.03 ^a^	NS	0.04 ^b^
T8	0.02 ^a^	0.03 ^a^	NS	NS
O1	0.02 ^a^	0.01 ^a^	NS	NS
O2	0.02 ^a^	0.04 ^a^	NS	NS

^a^ Significantly higher values obtained against the easier opponent. ^b^ Significantly higher values obtained against the most difficult opponent.
